# Editorial: The Role of Biomarkers in the Immunopathology and Diagnosis of Immune Exacerbations in Leprosy—New Frontiers to Manage This Neglected Disease

**DOI:** 10.3389/fmed.2022.878781

**Published:** 2022-03-25

**Authors:** Paulo R. Z. Antas, Dilvani O. Santos

**Affiliations:** ^1^Laboratório de Imunologia Clínica, Instituto Oswaldo Cruz, Fiocruz, Rio de Janeiro, Brazil; ^2^Department of Infectious Diseases, Leiden University Medical Centre, Leiden, Netherlands; ^3^Programa de Pós-graduação em Ciências e Biotecnologia, Universidade Federal Fluminense, Rio de Janeiro, Brazil

**Keywords:** leprosy, biomarkers, reactional episodes, immune response, ENL, RR

Leprosy, also known as Hansen's disease, is a chronic, inflammatory, debilitating, stigmatized, and neglected infectious disease caused by the obligate intracellular parasite *Mycobacterium leprae* (also referred as *M. lepromatosis* in Central America), and it has existed throughout recorded history. Clinical manifestations in leprosy primarily depend on the host's immune response profile to the pathogen. At one end of the spectrum lies paucibacillary tuberculoid patients (TT forms) with a single skin lesion, and *in vitro* and *in vivo* cell-mediated immune (CMI) responses (Figure 1). On the other hand, multibacillary lepromatous patients (LL) reveal numerous skin lesions, and absence of specific CMI responses. During the course of leprosy, a reasonable proportion of patients (30–50%) will develop reactional, mostly severe episodes of acute inflammation, imposing a major clinical challenge to practitioners in the management of leprosy patients. These events have effects on the skin and nerves, are accompanied or not by systemic symptoms, and are classified as type I (reversal reaction, RR) or type II (erythema nodosum leprosum, ENL) reactions depending on the clinical characteristics of the acute inflammation and its immunological background (Figure 1). In addition to these, the Lucio phenomenon is a rare complication characterized by an acute and severe necrotizing vasculitis, accompanied by profound anemia, requiring intensive monitoring. It has been long speculated that these reactions may occur during leprosy development when a patient shows increased CMI response against *M. leprae*, and the patient's clinical status could move toward the TT form (Figure 1). Those reactional episodes are frequently detected simultaneously with leprosy, but more often arise several months after the treatment onset (2).

**Figure 1:**
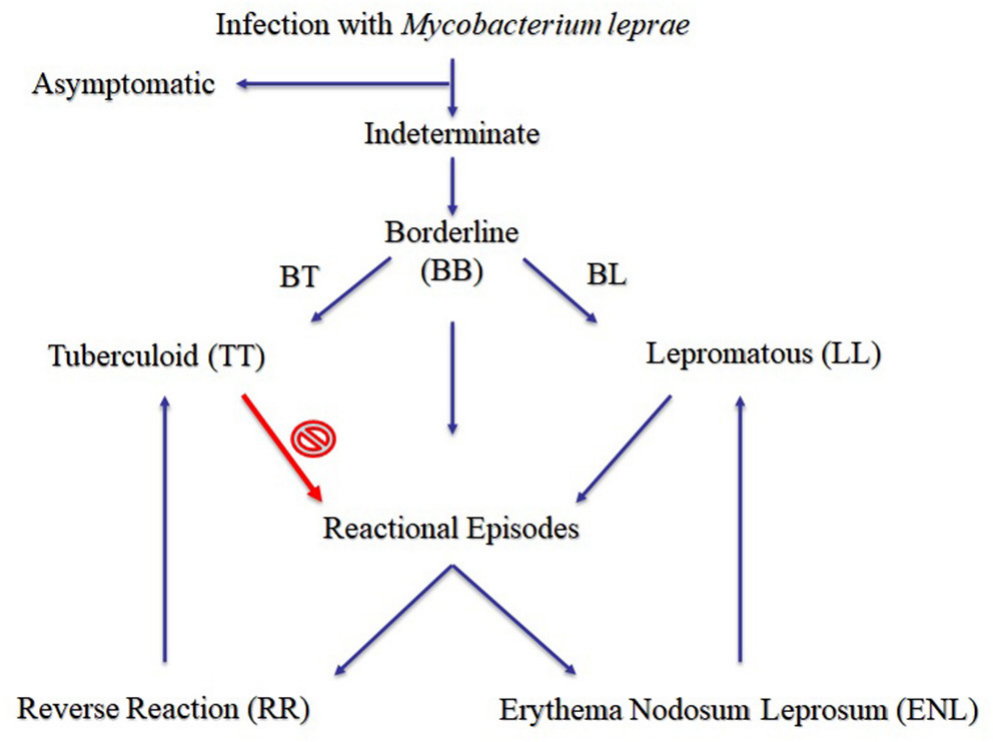
Proposed clinical forms of leprosy (1).

In light of the occurrence of the acute inflammatory responses, reactions are considered a clinical emergency and anti-inflammatory treatment must be promptly initiated. ENL patients are treated preferentially with thalidomide (300 mg/day), or pentoxifylline (a methyl-xantine derivative and known TNF inhibitor), associated or not with steroids. One way or another, future research efforts must be directed toward the discovery of new biomarkers for early detection of ENL episodes, in order to prevent physical disabilities and additional *M. leprae* transmission.

It is with this in mind that this Frontiers in Medicine Research Topic-specific collection of articles was conceptualized to present (i) new findings in order to keep readers up to date regarding those reactional episodes and (ii) to provide a forum to debate what is currently known about leprosy biomarkers research. Hence, the five outstanding articles published on that scope were mainly focused on the clinical and experimental data that looked at the immunopathology of both RR and ENL episodes, and to the susceptibility of the patients that developed such severe inflammatory reactions.

Luo et al. reviewed the progress of immunological, genetical, transcriptional, and metabolical biomarkers research for the pathogenesis of leprosy reactions, highlighting the ones linked to dysfunction and inflammation. The authors confirmed that no biomarkers have yet been found to reliably predict the occurrence of leprosy reactions, particularly to distinguish RR from ENL patients. Prior assessment has suggested that leprosy is a genetic disease: Several immune-related genes of hosts have long been considered major contributors due to its association with leprosy (3). Sahu et al. also reviewed the potential related to the abundance of circulating neutrophils, particularly the metabolic reprogramming of those *M. leprae*-infected cells secreting/excreting exosomes, as diagnostic and prognostic biomarker tools. In 2010, a pivotal study found that the human tuberculosis blood-transcriptional signature was dominated by a neutrophil-driven interferon-inducible gene profile through modular and pathway analysis (4). Yet, and since the first report in 1986 of a subpopulation of circulating low-density neutrophils (LDNs) being described and linked to autoimmune diseases (5), LDNs have also been associated with the pathogenesis and severity of COVID-19, thrombocytopenia, myopathies, psoriasis, cancer, sepsis, and tuberculosis. Forasteiro Tavares et al. found those LDNs with an activated phenotype in the blood of ENL patients as well. Interestingly, those ENL patients under thalidomide therapy also showed a similar frequency of LDNs as observed before treatment. In addition, the MMP-9 serum levels in LL patients with or without ENL episodes correlated well with LDN frequency. That study also uncovered an *in vitro* immunomodulation of LDNs by *M. leprae* and TGF-β.

Once ENL episodes had been strongly associated with higher TNF production, several studies were performed to determine whether pentoxifylline was able to ameliorate the clinical symptoms of ENL by modulating TNF levels (6). Accordingly, treatment of reactional ENL patients with pentoxifylline decreased TNF levels in the sera and also inhibited *in vitro M. leprae*-induced TNF production. In the skin biopsies of patients before and during the onset of those type II reactions, gene expression assessment showed upregulation of TNF, IL-6, and IFNγ mRNA (7). Bi-directional dose-dependent TNF regulation has been demonstrated by several groups, depending on cell type and method of cellular activation. Thus, in regard to reactional ENL patients who were under management by dose variation of thalidomide and prednisone usages, genetic associations were found by Maciel-Fiuza et al. for TLR1, TLR2, TLR4 (only thalidomide though), and TLR6 in a time-dependent manner. Actually, the impact of ethnic background has been long suspected of contributing to a host-genetic risk factor for developing those reactional episodes (2). This is discussed in more detail in this issue where TLR polymorphisms might play an important role during the response to ENL treatment. Conversely, authors argued that those polymorphisms could not be considered as useful biomarkers in the clinical setting due to small differences in medication doses.

Seminal papers have already shown that serum IgM, IgG, and IgA levels were significantly raised after subsidence of ENL episodes (8). This is revisited in this issue where Silva et al. have now shown specific, high serum IgA levels in LL, but not in TT patients. In fact, the latter showed increased IgA levels against a naturally conjugated antigen (NDO-HAS). Also, all household contacts of a leprosy patient displayed higher IgA reactivity to NDO-HSA than the non-endemic controls, which give rise to the possibility for its potential utility in household contact follow-up in leprosy endemic settings.

It is becoming increasingly clear that the action of any individual soluble biomarker is neither completely beneficial nor totally detrimental to the host. Rather, a fine-tune balance of a given biomarker production and regulation must be maintained to ensure that the host can effectively react to invading microorganisms without compromising host wellbeing in the process. And this also seems to be true for *M. leprae* infections. A better understanding of the regulation of the network of cells and cytokines will provide us with a rational approach to design interventions to modulate the host's immune response to *M. leprae* infection and prevent damaging immune-mediated pathologies, such as the reactional episodes. At the same time, biomarkers research may uncover whether true interventions will perform better than the usual leprosy management.

## Author Contributions

DS made a central conceptualization of this research topic. PA contributed original draft preparation of this editorial. PA and DS contributed in final review and editing. Both authors listed have made a substantial, direct, and intellectual contribution to the work and approved it for publication.

## Funding

This work was partly supported by IOC/FIOCRUZ. PA is granted with a PrInt Fiocruz-CAPES Program for senior visiting professor scholarship.

## Conflict of Interest

The authors declare that the research was conducted in the absence of any commercial or financial relationships that could be construed as a potential conflict of interest.

## Publisher's Note

All claims expressed in this article are solely those of the authors and do not necessarily represent those of their affiliated organizations, or those of the publisher, the editors and the reviewers. Any product that may be evaluated in this article, or claim that may be made by its manufacturer, is not guaranteed or endorsed by the publisher.
